# A machine learning approach to investigate the role of fear of pain, personal experience, and vicarious learning in dental anxiety

**DOI:** 10.1186/s12903-025-05973-9

**Published:** 2025-04-18

**Authors:** Andras N. Zsido, Botond Laszlo Kiss, Carlos M. Coelho, Andras Matuz, Pooya Pasandideh Rahvard, Bela Birkas

**Affiliations:** 1 Institute of Psychology, University of Pécs, 6 Ifjusag Street, Pécs, Baranya H 7624, Pécs, Hungary; 2 Research Centre for Contemporary Challenges, University of Pécs, Pécs, Hungary; 3 Szentágothai Research Centre, University of Pécs, Pécs, Hungary; 4 Department of Psychology, University of the Azores, Ponta Delgada, Portugal; 5 University Research Center in Psychology (CUIP), University of Azores, Ponta Delgada, Portugal; 6 Medical School, University of Pécs, Pécs, Hungary; 7 Damira Dental Studios, London, UK

**Keywords:** Odontophobia, Dental fear, Injection, Exposure, Experience, Social transmission

## Abstract

**Background:**

Dental anxiety is a pervasive problem worldwide, leading to avoidance of dental care, resulting in oral health problems and impacting daily life through social withdrawal and work absenteeism. Addressing this fear is an important public health concern. This study aimed to identify factors that are negatively (e.g., regular dental visits) and positively (e.g., receiving injections, social transmission of fear, anxiety) associated with dental anxiety.

**Methods:**

We examined the relationship between dental anxiety and state anxiety, fear of pain associated with injections and other bodily harm, personal experience, and social transmission of fear using a machine learning approach. Participants (*N* = 802, 625 women, M age = 28.74, SD = 9.15) completed an online survey.

**Results:**

Results showed that higher regularity of dental visits was negatively associated (coefficient range: − 0.055 to − 0.097) with dental anxiety. Conversely, we found positive associations between higher levels of anxiety (coefficient range: 0.130 to 0.168), subjectively more painful and distressing last dental visit (coefficient range: 0.322 to 0.343), social transmission of fear (hearing a scary story from friends [coefficient range: 0.024 to 0.128] or the media [coefficient range: 0.024 to 0.027]), and higher fear of pain associated with injections (coefficient range: 0.020 to 0.260) and dental anxiety.

**Conclusions:**

These findings highlight the important role of fear of pain associated with injections in dental anxiety, and support that prior benign exposures are protective, while negative experiences increase the risk of developing dental anxiety. Our findings are also consistent with social transmission theories.

## Introduction

Despite significant advances in dentistry, including pain control, anesthesia, instruments, techniques, and patient management, dental anxiety remains a significant problem for both patients and dentists [1, 2]. Many people still fear and openly admit their fear of dental services [3]. Dental phobia affects between 3% and 5% of adults in Western countries, with up to 40% expressing fear of dental procedures [4, 5]. A systematic review and meta-analysis of 31 publications that included a sample of 72,577 adults aged 18 years and older [6] showed that the global estimated prevalence of dental fear and anxiety^1^ was 15.3% (95% CI 10.2–21.2), with high anxiety at 12.4% (95% CI 9.5–15.6) and severe anxiety at 3.3% (95% CI 0.9–7.1), indicating that dental anxiety is prevalent among adults worldwide.

Dental anxiety poses a significant challenge to patient management, as anxious individuals are more likely to avoid, postpone, or cancel dental appointments [1, 7, 8]. Indeed, it has been observed that those who experience oral health problems are more likely to report severe dental anxiety, resulting in infrequent visits and chronic appointment cancellations [9, 10]. Consequently, anxious patients often have more decayed teeth and fewer fillings [3, 11] because dental anxiety leads to treatment avoidance, which affects oral health and the quality of dental care. This avoidance extends into daily life, often resulting in difficulties in social interactions, work absenteeism, feelings of shame, and social withdrawal [4, 12]. These effects create a *vicious cycle* [13] in which anxiety is reinforced, leading to avoidance of care, which in turn leads to deteriorating oral health and increased reliance on medication, which increases both the need for treatment and further anxiety. On the other hand, reducing dental anxiety is associated with improved mood and reduced dependence on tranquilizers and alcohol, among other benefits [12]. As such, reducing this fear is a relevant public health issue, as it appears to be a fairly ubiquitous problem [14, 15].

The administration of local anesthetics is often perceived as the most painful aspect of medical or dental procedures, and fear of injections has been identified as a significant reason why patients avoid dental care [16, 17]. Concerns about potential pain during dental visits– often revolve around the administration of injections and the use of dental drills [18, 19]– are thought to play a key role in the development and maintenance of dental anxiety [20]. Patients with dental anxiety report experiencing more intense and prolonged pain than their less anxious counterparts [21]. A well-known study [22] surveyed students and staff at the University of Washington regarding avoidance of dental care and fear of dental injections. More than 25% of them reported fear of injections, and nearly 5% reported avoiding, canceling, or not attending at least one dental appointment because of fear of dental injections. Avoiders rated all aspects of their last injection as significantly more anxiety-inducing, painful, and unpleasant than non-avoiders. Avoidance of dental screening and treatment can lead to more difficult and painful procedures later, which further increases anxiety and promotes subsequent avoidance behaviors [13]. These often require additional techniques to ensure pain management, and failure to achieve adequate anesthesia can hinder the clinician’s ability to perform successfully [23]. Thus, fear of the pain associated with injections appears to be a driving factor in dental anxiety.

Pain is influenced by prior experiences, anticipation, and the level of anxiety [24]. For instance, a study [25] found a significant correlation between the perceived pain of previous dental treatments and the reporting of these experiences as traumatic, both of which were associated with higher levels of dental anxiety. On the other hand, there is also evidence to support the latent inhibition hypothesis. Latent inhibition refers to the phenomenon whereby pre-exposure to a neutral stimulus impairs the subsequent learning of an association between that stimulus and a biologically salient event. In the context of fear and anxiety, this suggests that the more experience an individual has with a stimulus in a non-threatening context, the less likely they are to develop an intense fear response or phobia towards that object. This phenomenon highlights the protective role of prior neutral exposure in reducing the likelihood of fear acquisition [15]. Individuals are less likely to develop dental anxiety if they have undergone several relatively painless treatments prior to conditioning [25, 26]. This (traumatic) conditioning refers to the process by which an individual develops a fear or anxiety response through direct exposure to a traumatic event [25–27]. In this process, a neutral stimulus (e.g., dental office) is associated with an aversive or distressing experience (e.g., pain), resulting in the conditioned stimulus eliciting fear or anxiety even in the absence of the original traumatic event. Conditioning experiences are a key mechanism in the development of specific fears and phobias, where an intense emotional response is learned and subsequently triggered by reminders of the original event. Past studies [24, 28] found that prior distressing dental experiences and preoperative anxiety levels significantly influenced anxiety levels at the four-week follow-up, accounting for 71% of the observed variation. Another study [26] examined the factors that distinguish individuals with mild and severe dental anxiety from those without. Results showed that participants who had never experienced dental anxiety were less likely to have undergone painful dental procedures than those who reported anxiety. In addition, individuals who experienced pain during dental treatment but did not develop anxiety had a history supporting a latent inhibition effect. This is consistent with previous findings suggesting that greater knowledge and prior experience can reduce the likelihood of avoiding medical situations [7, 29]. These findings have implications for treatment strategies and highlight the need to address these contextual factors in prevention efforts to encourage regular screening and timely medical care.

In addition to direct personal experiences, fear can also develop through indirect processes [30]. Social transmission of fear or social learning (which can be subdivided into vicarious fear learning and information learning) plays an important role in shaping emotional responses [31, 32]. Vicarious learning involves learning fears by observing the reactions of others, while information learning means hearing frightening stories from peers and friends, or being exposed to frightening information through media channels [33, 34]. While fear is primarily influenced by expectations of dental trauma and previous painful experiences [18], approximately 17% of individuals also reported that stories shared by friends, family, and the media influenced their expectations of trauma related to dental visits. Although social learning mostly occurs in childhood [35–37], there are some prior studies that indicate the possibility of these types of learning in adulthood [34, 38]. However, most studies to date have focused only on the transmission of dental anxiety from parents to children [39, 40]. As a result, less is known about exactly how stories heard from friends and through the media affect dental anxiety.

Our overall goal in the present study was to examine the effects of anxiety, fear of pain associated with injections and other bodily harm, personal experience, and social learning on dental anxiety. To prevent the serious health and social consequences of dental anxiety, it is important to identify relevant risk factors for avoidance. Previous studies often focused on only one of these factors, failing to provide a comprehensive understanding of their relative importance. Specifically, based on former findings, we hypothesized that injection-related pain (but not other bodily harm) and distress at the last visit would emerge as the most important risk factors for dental anxiety. In addition, a higher exposure rate, i.e., more frequent visits to the dentist and longer treatments, would be negatively associated with the development of severe dental anxiety. For this purpose, we utilized a machine learning approach that inherently performs feature selection simplifying the model, and focusing on the most important variables, providing robust and interpretable importance scores. By combining these features, our analysis ensured a more reliable and parsimonious model compared to conventional regression, thereby increasing the robustness and generalizability of our findings.

## Methods

### Participants

We determined the minimum sample size required by calculating the estimated statistical power for linear regression with a small effect size and a conservative approach (f^2^=0.05, β > 0.95, alpha = 0.05) using the G*Power 3 software [41]. The analysis indicated a minimum required sample size of 543. However, we aimed to recruit more participants to allow for a machine learning approach to data analysis and greater variability in participants. We recruited 802 participants (625 women) between the ages of 18 and 72 years (M = 28.74, SD = 9.15).

All participants were recruited via the Internet by posting invitations on various social media forums and mailing lists in order to obtain a non-clinical, heterogeneous sample. None of the subjects reported having been diagnosed with a specific phobia by a physician or psychiatrist. Subjects participated voluntarily. The research was approved by the Hungarian United Ethical Review Committee for Research in Psychology and was conducted in accordance with the World Medical Association’s Code of Ethics (Declaration of Helsinki). Written informed consent was obtained from all participants.

The survey and the dataset supporting the conclusions of this article are available in the Open Science Framework repository, https://osf.io/n2hu5/.

### Questionnaires

#### Sociodemographic questions

Sociodemographic data included age and sex, questions about previous personal experiences (frequency of dental visits, serious dental problems as a child or adult, treatment lasting more than six months) and social learning including vicarious exposure and transmission of information through social channels (having heard frightening stories, distressing information in the media, witnessing a treatment) about dental practice. We also asked participants to rate how painful or distressing their last visit to the dentist was on a five-point scale (from 0 - not at all to 5 - extremely).

#### Dental anxiety

We measured dental anxiety (and fears) with three separate questionnaires. The use of three separate questionnaires to measure dental anxiety strengthens the study by providing a more comprehensive assessment of the construct and increases the reliability of the results through triangulation. In addition, this approach allows for greater generalizability of the findings by taking into account the differences in how dental anxiety may manifest itself in different measurement tools.

The first was the Dental Anxiety Question (DAQ) rated on a three-point scale [42]. The original study of the DAQ reported 93% agreement between the single-item question and the Modified Dental Anxiety Scale (see details below); the kappa coefficient was 0.63, the specificity was 0.95, and the sensitivity was 0.80, suggesting that it has good validity and is a psychometrically sound measure. The second was the Short Dental Fear Question (SDFQ) rated on a four-point scale [43]. The original study of the SDFQ reported Spearman correlations between the SDFQ and other, longer measures of dental anxiety ranging from 0.69 to 0.79, suggesting that the SDFQ captures the essence of these other instruments and may be suitable for measuring dental anxiety. Both the DAQ and the SDFQ are single-item questionnaires. The third was the Modified Dental Anxiety Scale (MDAS), a five-item questionnaire that evaluates the emotional reactions of the respondents to different situations (e.g., waiting, scaling) during a dental visit [44]. Items were rated on a five-point scale. In the original study of the MDAS, the Kaiser-Meyer-Olkin value was 0.842, indicating the presence of sufficient common variance to merit factor analysis, and the eigenvalue of 3.69 showed a clear unidimensional factor structure and that the scale could be considered unidimensional. The goodness of fit statistics showed excellent agreement between the model and the raw data (x^2^ = 3.89; CFI = 0.999, TLI = 0.997, RMSEA = 0.031). The internal consistency coefficient of the scale was excellent (0.957, and the 95%CI being 0.953– 0.961). The McDonald’s omega for MDAS in the present study was 0.89. For all three measures, higher scores meant greater dental anxiety.

#### Fear of pain

We used the brief Fear of Pain Questionnaire (FPQ) to assess pain-related fear and anxiety about pain [45]. The participants rated the nine items on a five-point scale. We calculated two sum scores, one for items (nr. 4 and 8) related to injections and one for items (nr. 1, 2, 3, 5, 6, and 9) not associated with injections. Higher scores indicated higher levels of fear in both cases. In this study, McDonald’s omegas were 0.6 and 0.75.

#### State anxiety

We used a one-item question to measure the anxiety level of the participants [46]. The item was rated on a five-point scale, with higher scores meaning a higher anxiety level. We measured state anxiety to control for its potential confounding effect.

### Statistical analyses

We used a machine learning approach to explore the predictive values of socio-demographic factors and questionnaires on dental anxiety scores. All programming was implemented in Python, using the scikit-learn package [47]. The same analysis was conducted separately for all three dental anxiety questionnaires (DAQ, SDFQ, and MDAS). Least absolute shrinkage and selection operator (LASSO) regression was used with leave-one-out cross-validation on all three outcome variables. To account for statistical fluctuations, while assessing the impact of predictor variables, importance scores were obtained using permutation feature importance (*n* = 100). While conventional regression analysis could have been used, the use of LASSO regression offered several advantages as it inherently performs feature selection by setting the coefficients of less relevant predictors to zero, simplifying the model, and focusing on the most important variables. This approach reduces the risk of overfitting. In addition, the use of permutation feature importance allowed us to quantify the contribution of each predictor while accounting for statistical variation, providing robust and interpretable importance scores. By combining these features, our analysis ensured a more reliable and parsimonious model compared to conventional regression, thereby increasing the robustness and generalizability of our findings.

Accordingly, for each predictor variable, we present a mean importance value, an SD (the standard deviation between permutations), and a coefficient (standardized beta). We also display model performance as an R^2^ value (extracted from the cross-validation). Statistical results will be presented in tables instead of in the text to make the description of the results easier to follow and more understandable.

## Results

Table 1 shows the central tendencies of the questionnaires and more details about the sample.

**Table 1:** Detailed descriptive statistics of the sample, including demographic variables and questionnaires used in the study

	Median	Mean	SD	Min	Max
Age	26	28.74	9.15	18	72
Anxiety	2	2.14	1.14	1	5
Pain					
- not injection related	21	20.34	4.68	7	32
- injection related	5	5.30	1.85	2	10
MDAS	12	12.41	4.89	5	25
SDFQ	2	1.75	0.71	1	4
DAQ	2	1.68	0.64	1	3
Last visit pain & distress	2	2.423	1.171	1	5
	**Category**	**Count**	**Percent**		
Sex	Women	625	77.93%		
Checkup regularity	Never	77	9.60%		
	Few years	338	42.15%		
	Year	266	33.17%		
	Half year	121	15.09%		
Serious problem					
- as an adult	Yes	606	75.56%		
- as a child (< 12yo)	Yes	362	45.14%		
Prolonged treatment	Yes	211	26.31%		
Frightening story	Yes	372	46.38%		
Media distress	Yes	201	25.06%		
Witness treatment	Yes	238	29.68%		

Note: DAQ = Dental Anxiety Question, SDFQ = Short Dental Fear Question, MDAS = Modified Dental Anxiety Scale

**Fig. 1:**
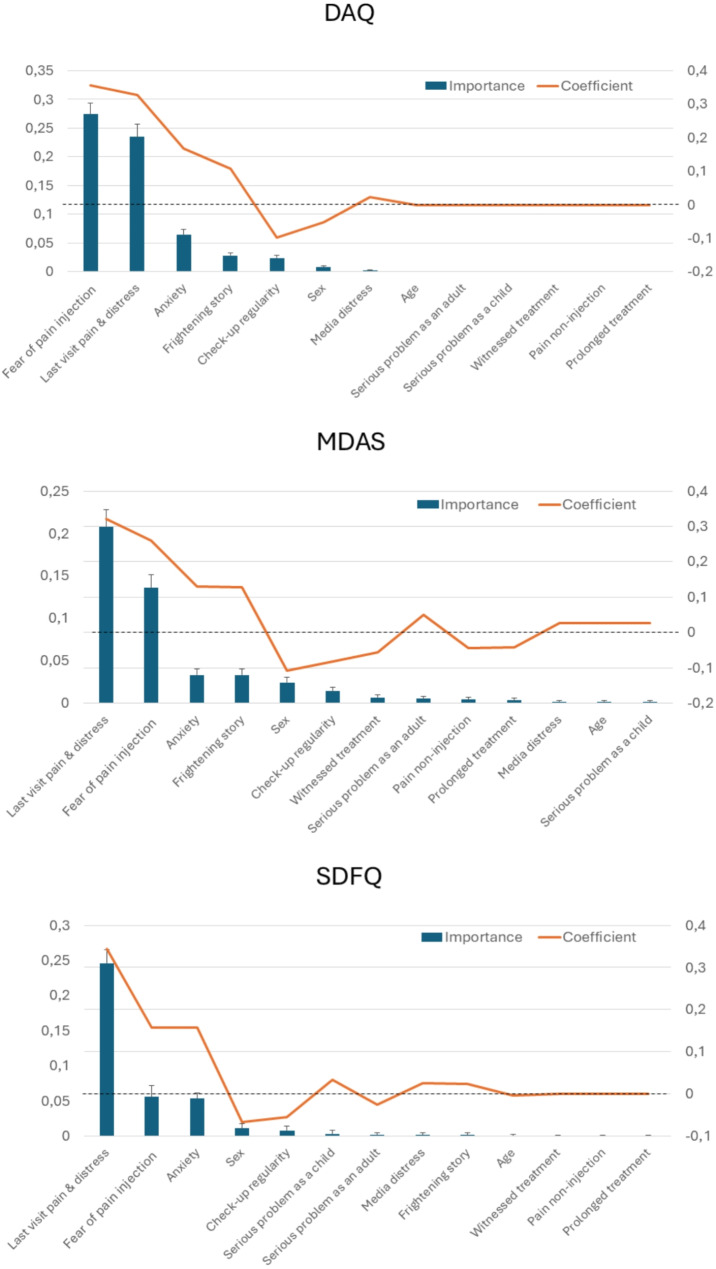
Results of feature selection based on least absolute shrinkage and selection operator regression predicting Dental Anxiety Question (DAQ), Modified Dental Anxiety Scale (MDAS), and Short Dental Fear Question (SDFQ) scores. Error bars represent the standard deviation of importance across 100 iterations

The first model (with DAQ) showed an adequate performance (R^2^ = 0.35). Figure 1; Table 2 shows the detailed statistical results. The strongest predictors were pain and distress reported regarding the last visit and fear of pain related to injections; both were positively associated with dental anxiety. Anxiety, having heard frightening stories or distressing information in the media, having a serious dental problem during childhood and adulthood, and age were also positive predictors. Negative predictors were higher check-up regularity, being male (compared to female), witnessing a treatment, fear of pain not related to injections, and having had a prolonged treatment.

The second model (with MDAS) showed a good performance (R^2^ = 0.50). See Fig. 1; Table 2 for the detailed statistical results. The strongest predictors were pain and distress reported regarding the last visit and fear of pain related to injections; both were positively associated with dental anxiety. Anxiety, and having heard frightening stories or distressing information in the media were also positive predictors. Negative predictors were higher check-up regularity and being male (compared to female). The other variables were left out of the model (i.e., their coefficients were set to zero by the LASSO regression).

**Table 2:** Detailed statistical results of the three tested models with the mean importance (Imp), standard deviation (SD), and coefficient (Coeff) values displayed for all variables

Feature	Model 1 - DAQ				Model 2 - SDFQ				Model 3 - MDAS		
	Imp	SD	Coeff		Imp	SD	Coeff		Imp	SD	Coeff
Age	0	0	0		0	0	-0.004		0.001	0.001	0.026
Sex^a^	0.008	0.002	-0.052		0.011	0.004	-0.067		0.024	0.006	-0.108
Check-up regularity	0.024	0.005	-0.097		0.008	0.003	-0.055		0.014	0.005	-0.083
Serious problem as adult^b^	0	0	0		0.002	0.002	-0.026		0.005	0.003	0.051
Serious problem as child^b^	0	0	0		0.003	0.002	0.033		0.001	0.001	0.027
Anxiety	0.065	0.009	0.168		0.054	0.009	0.158		0.033	0.007	0.130
Last visit pain & distress	0.235	0.021	0.329		0.246	0.026	0.343		0.208	0.020	0.322
Frightening story^b^	0.028	0.005	0.108		0.002	0.001	0.024		0.033	0.007	0.128
Media distress^b^	0.003	0.001	0.024		0.002	0.002	0.025		0.002	0.002	0.027
Witnessed treatment^b^	0	0	0		0	0	0		0.007	0.003	-0.057
Pain non-injection	0	0	0		0	0	0		0.004	0.003	-0.045
Pain injection	0.274	0.020	0.357		0.056	0.009	0.158		0.136	0.016	0.260
Prolonged treatmen^b^	0	0	0		0	0	0		0.003	0.002	-0.042
*Model performance*	*0.503*				*0.285*				*0.352*		

Note: ^a^ reference group: women; ^b^ reference group: no

DAQ = Dental Anxiety Question, SDFQ = Short Dental Fear Question, MDAS = Modified Dental Anxiety Scale

The third model (with SDFQ) showed adequate performance (R^2^ = 0.28). Figure 1; Table 2 shows the detailed statistical results. Here, the strongest predictor was, again, pain and distress reported with respect to the last visit, followed by anxiety and fear of pain related to injections. All three were positively associated with dental anxiety. Having a serious dental problem during childhood, and hearing frightening stories or distressing information in the media were also positive predictors. Negative predictors included age, being male, check-up regularity, and having had a serious dental problem as an adult. The other variables were left out of the model.

## Discussion

Our study aimed to identify the factors positively and negatively associated with dental anxiety. We used three questionnaires to measure dental anxiety to strengthen our findings, provide a more comprehensive assessment of the construct, and allow for greater generalizability. Our hypothesis that injection-related pain (but not other bodily harm) and distress at the last visit emerged as the most important factors positively associated with dental anxiety was supported by the results. Our second hypothesis that a higher exposure would be negatively associated with the development of severe dental anxiety was only partly supported because only more frequent visits to the dentist showed such an effect. Our findings are consistent with previous studies that have highlighted the importance of fear of injections, personal experiences, and social factors in the aetiology of dental anxiety; and that addressing these causes is important to reduce anxiety.

Our results showed that fear and pain associated with injections are an important part of dental anxiety, consistent with the findings of previous studies [16, 18, 19, 22, 48]. It is sometimes difficult to know which specific elements of the medical procedure are associated with fear acquisition and avoidance [49]. Pain may or may not be associated with injections, and injections may cause anxiety in addition to pain. A study [24] with patients undergoing oral surgery showed that patients anticipated significantly more pain than they actually experienced with regard to a mandibular block injection. Fear of injection pain is the most common dimension of dental anxiety [22]. Therefore, dentists should focus not only on the effectiveness of pain control but also on the patient’s experience of pain, pressure, and discomfort associated with the injection itself [16, 17, 48]. This may help to reduce the discomfort and distress associated with dental visits (or the anticipation of treatment), which is another important factor according to our findings.

We found support for the notion that previous negative experiences are a risk factor [25, 26], whereas benign exposures are protective [7, 24, 28, 29] regarding dental anxiety. Pain and distress experienced during the last dental visit were strongly related to the intensity of dental anxiety participants felt. As noted in previous studies, dental anxiety (and general anxiety) has a consistent impact on pain throughout the entire period of dental treatment [50], as anxious patients expect more pain [24] and are consequently more likely to postpone, avoid or cancel their appointments [7, 13]. This can have serious consequences, because if they do attend the treatment, they are likely to perceive it as more painful, which increases their fear and anxiety, leading to future avoidance of dental visits. Avoidance learning is a fundamental process in the development and maintenance of fears [27, 51]. Although avoidance may temporarily reduce discomfort, it often perpetuates and reinforces anxiety by preventing exposure to corrective experiences that might disconfirm the perceived threat.

In fact, it has been shown that individuals report less fear when they have personal experience with a treatment and know what to expect [52]. Furthermore, repeated previous experiences of real-life situations can give people a sense of control over them and thus reduce their anxiety [53], possibly through a habituation or fear inoculation effect [54, 55]. However, it was the frequency of visits that had a protective effect, promoting positive learning experiences and latent inhibition. More frequent visits suggest that the treatments required were likely to be minor, such as routine check-ups, examinations, scaling and polishing or simple restorations. In contrast, longer treatments do not necessarily indicate atraumatic visits, as they often involve more complex and challenging procedures such as endodontics, crown and bridge preparation, surgical extractions, or extensive treatment needed to restore oral health in patients who have not had regular dental care. Although this study assessed general fear of pain, our primary focus was on injection-related pain due to its stronger association with dental anxiety as reported in previous research; however, we acknowledge the importance of exploring the broader relationship between general fear of pain and dental anxiety in future studies. Promoting regular check-ups is beneficial for both patients and dentists as it prevents the development of more serious health problems.

Our findings that fear can also be acquired by social information learning, i.e. hearing frightening stories are consistent with social transmission theories [33, 34]. Although the first studies [18] on dental anxiety noted that a significant proportion of respondents reported that such stories heard from peers, families or the media negatively influenced their expectations of pain and distress associated with dental visits, to our knowledge no study has focused on this aspect of dental anxiety acquisition in adults. Our findings are consistent with studies focusing on the transmission of dental anxiety from parents to children [39, 40]. Emotional transmission of dental anxiety is possible not only between family members, but also in much wider settings, such as peer groups or even through the media. Considering this finding, future studies are required to assess whether positive social information learning is also possible, i.e., positive stories from peers and the media highlighting that dental visits can be completely painless (including the administration of local anesthetics) and that regular check-ups significantly reduce the likelihood of needing more serious and prolonged treatment could lead to a reduction in dental anxiety.

Although our study provides valuable insights into the predictors of dental anxiety, some limitations should be acknowledged. First, the cross-sectional design limits causal inference; we cannot determine whether the identified predictors are causes or consequences of dental anxiety. Second, our reliance on self-reported measures may introduce recall or social desirability biases, particularly in reporting past experiences or levels of fear. Third, the majority of our sample consisted of women who are, according to our results and previous studies more prone to experience dental anxiety. While this means the results are true for people at risk, our results might not be valid regarding the effects of sex differences. Fourth, although we used three validated questionnaires to increase generalizability, the results of the study may not fully capture cultural (e.g. beliefs) or contextual (e.g. socioeconomic, economic, trust) differences in dental anxiety in different populations. Finally, we did not specify between childhood/adolescence and adulthood in terms of social learning. We decided to include only general questions because most of them occur in childhood, although there is a possibility that they may occur later in life.

## Conclusions

In conclusion, our study provides a nuanced understanding of the predictors of dental anxiety, with implications for both clinical practice and public health. By addressing both individual and social factors, targeted interventions may help to reduce dental anxiety, improve patient experience, and promote better oral health outcomes. The findings highlight the importance of reducing pain and distress during dental procedures, with particular attention to patients’ emotional response to injections. Dentists should adopt approaches that not only ensure effective pain control, but also address the sensory and emotional components of treatment to improve patient comfort and reduce anxiety. In addition, encouraging regular dental visits and positive dental experiences can promote habituation, build a sense of control, and counteract avoidance behaviors that perpetuate anxiety. Public health campaigns could aim to replace negative narratives about dental treatment with positive, reassuring messages, particularly through media channels. Future research should explore whether the promotion of positive emotional messages could actively reduce dental anxiety and its associated public health burden.

## Data Availability

The survey and the dataset supporting the conclusions of this article is available in the Open Science Framework repository, https://osf.io/n2hu5/.

## Footnotes

1 Since the MeSH term dental anxiety also includes dental fear, henceforth we will refer to them as dental anxiety.

## Abbreviations

CFI: Comparative Fit Index
DAQ: Dental Anxiety Question
FPQ: Fear of Pain Questionnaire
LASSO: Least absolute shrinkage and selection operator
MDAS: Modified Dental Anxiety Scale
RMSEA: Root Mean Square Error of Approximation
SD: Standard deviation
SDFQ: Short Dental Fear Question
TLI: Tucker-Lewis Index
